# Preparation and Characterization of Silicon-Metal Fluoride Reactive Composites

**DOI:** 10.3390/nano10122367

**Published:** 2020-11-28

**Authors:** Siva Kumar Valluri, Mirko Schoenitz, Edward Dreizin

**Affiliations:** 1 O.H. York Department of Chemical and Materials Engineering, New Jersey Institute of Technology, Newark, NJ 07102, USA; sv476@njit.edu (S.K.V.); schoenit@njit.edu (M.S.); 2 High-Energy and Special Materials Research Laboratory, Tomsk State University, 634050 Tomsk, Russia

**Keywords:** reactive materials, nanocomposite, metal combustion, ignition, thermal analysis

## Abstract

Fuel-rich composite powders combining elemental Si with the metal fluoride oxidizers BiF_3_ and CoF_2_ were prepared by arrested reactive milling. Reactivity of the composite powders was assessed using thermoanalytical measurements in both inert (Ar) and oxidizing (Ar/O_2_) environments. Powders were ignited using an electrically heated filament; particle combustion experiments were performed in room air using a CO_2_ laser as an ignition source. Both composites showed accelerated oxidation of Si when heated in oxidizing environments and ignited readily using the heated filament. Elemental Si, used as a reference, did not exhibit appreciable oxidation when heated under the same conditions and could not be ignited using either a heated filament or laser. Lower-temperature Si fluoride formation and oxidation were observed for the composites with BiF_3_; respectively, the ignition temperature for these composite powders was also lower. Particle combustion experiments were successful with the Si/BiF_3_ composite. The statistical distribution of the measured particle burn times was correlated with the measured particle size distribution to establish the effect of particle sizes on their burn times. The measured burn times were close to those measured for similar composites with Al and B serving as fuels.

## 1. Introduction

Silicon serves as a fuel in several pyrotechnic compositions [1]. Its high energy density, abundance in the lithosphere [2] and cheap manufacturing make it an attractive fuel for a broad range of applications, including propellants and explosives. However, it is known to be relatively difficult to burn [3,4]. Silicon has a high boiling point (3538 K) compared to its oxide (2503 K) and hence burns heterogeneously. Further, the nascent refractory oxide layer on silicon particles limits the inward diffusion of oxygen at temperatures below 1273 K [5]. The direct oxidation of silicon from gaseous oxygen [6] and nitrogen [7] is only accelerated at higher temperatures, when diffusion rates increase.

To make silicon more reactive, previous work focused on employing aggressive condensed phase oxidizers. Transition metal oxides [4,8,9,10,11], alkali metal nitrates [4,9,10,11], perchlorates [9] and manganates [9] were explored as oxidizers for micron-sized and nanometric silicon powders. These compositions, though favorably reactive, do not fully exploit the potential of silicon as they yield largely condensed phase combustion products. Respective silicon-condensed oxidizer composites have reduced energy content compared to elemental silicon reacting with oxygen gas; thus, the reactions have reduced adiabatic flame temperatures. Use of high surface area, porous nanosilicon has attracted attention [9,12,13,14]; however, such materials may be difficult to handle and their applications are mostly limited to pyrotechnics.

In comparison to oxidation, fluorination of silicon is preferred in fuel-limited systems because the negative heat of formation of silicon tetrafluoride, SiF_4_, (−1615 kJ/mol) is substantially lower than that of SiO_2_ (−911 kJ/mol), making the reaction more exothermic. Silicon fluorides (and most oxyfluorides [15]) are gases under standard conditions.

Combustion-related work on fluorination of silicon was mostly limited to the use of fluoropolymers, e.g., polytetrafluoroethylene (PTFE) as oxidizers. Silicon-PTFE compositions are predicted to have a higher adiabatic combustion temperature and produce more gaseous products compared to combinations of silicon with oxide-based oxidizers [9]. Silicon-PTFE milled composites with a range of compositions (10–40 wt.% silicon) have been explored [16,17,18].

In recent studies it was shown that metal fluorides, such as CoF_2_, NiF_2_ and BiF_3_, could be effective oxidizers with metal fuels, e.g., aluminum. Reduced ignition temperatures and higher burn rates than for pure aluminum (in air) were reported for respective composites [19,20]. Further, metal fluorides were combined with boron, known to be more difficult to ignite than aluminum. A marked improvement in the ignition and combustion characteristics for boron/metal fluoride composites was observed with as little as 10 wt.% of BiF_3_ [21,22]. The addition of fluorides altered combustion of boron particles: it occurred in a single stage with shortened burn times [21,22,23]. In constant volume explosion experiments, the 10 wt.% BiF_3_ coated boron powders exhibited a higher rate of pressurization and higher maximum pressure compared to fine aluminum [22]. Experiments with aluminum and boron-based reactive composites containing metal fluorides as oxidizers show consistently significant improvements in kinetics of reactions leading to ignition [24,25].

Despite the increased reactivity, the fluoride composites made with aluminum and boron were found to be safer to handle, as they were insensitive to electrostatic stimulus unlike oxide based compositions [24,25]. It should also be noted that the gas-phase fluorinated reaction products could be aggressive; thus care must be taken when such products are released.

The use of metal fluorides as oxidizers for silicon is explored in the current work. The fluorides used in this study, BiF_3_ and CoF_2_ were selected because they are less hygroscopic, easy to handle [25], and contain metals that interact differently with metal fuels: Bi does not alloy with most metals or metalloids while Co forms alloys readily. The phase of the reduced metal (pure element or alloy) could affect further oxidation and combustion reactions. Based only on gravimetric and volumetric heats of reaction shown in Table 1, replacing oxides of Co and Bi with respective fluorides does not lead to tangible advantages. The heats of reactions are nearly matching for the metal-rich composites (with 50 wt.% of Si) reacting in presence of the external oxidizer, so that excess of Si and the metals obtained from the reduced fluorides can further be oxidized. Thus, the anticipated advantages of using metal fluorides are not based on the expected theoretical energy release but are based on the expected accelerated reaction rates and formation of gas phase products.

Both BiF_3_ and CoF_2_ were incorporated into silicon-metal fluoride composites prepared by arrested reactive milling [26].

## 2. Experimental

### 2.1. Materials Preparation

The silicon-metal fluoride composite powders were prepared in a SPEX Certiprep 8000 series shaker mill (by SPEX CertiPrep, Metuchen, NJ, USA). The constituent silicon (−325 mesh crystalline Si, 99%) and anhydrous fluoride powders, bismuth (III) fluoride (BiF_3_, 98%) and cobalt (II) fluoride (CoF_2_, 99%) were sourced from Alfa Aesar, Ward Hill, MA, USA. Preliminary inspection using electron microscopy showed that BiF_3_ contained crystalline particle agglomerates, mostly in the 10–15 μm range. For CoF_2_, the powder appeared as finer powder, with most particles in the single micron range. The compositions targeted 30 and 50 wt.% of silicon with the remaining mass of bismuth or cobalt fluoride. The materials were prepared in 5 g batches in steel milling vials. The powders were milled using steel milling media with hexane as the process control agent. The ball to powder mass ratio was fixed at 10.

In an initial, exploratory experiment, 50 wt.% silicon and 50 wt.% bismuth fluoride were milled for 60 min using 9.25-mm (3/8 in) diameter steel balls with 7 mL of hexane. This material is referred to as 1-stage 50Si·50BiF_3_. Examination of 1-stage 50Si·50BiF_3_ samples showed the presence of large Si particles that were not mixed with BiF_3_. To improve homogeneity, milling was therefore carried out it two steps for all other prepared composites. Silicon was milled separately in the first milling step to refine it. This step lasted 60 min and used 14 mL of hexane and 5-mm steel balls. This size-reduced silicon was also used as reference material for thermal analysis and other experiments.

In the second step, the size-reduced (premilled) silicon was milled with bismuth fluoride or cobalt fluoride for 60 min using 4.76-mm steel balls with 7 mL of hexane. These composites are referred to as 50Si·50BiF_3_, 50Si·50CoF_2_, 30Si·70BiF_3_ and 30Si·70CoF_2_, with respective mass percentages of components used as identifiers. Table 2 presents characteristics of the prepared materials. The equivalence ratios describe reactions.

(1) $$Si+\frac{4}{x}MeF_{x}\to SiF_{4}↑+\frac{4}{x}Me$$

where Me stands for Bi or Co. Reactions (1) reduce the respective metal fluorides and yield SiF_4_. All prepared composites are fuel-rich, making them potential fuel candidates for applications where oxygenated gas environments are available.

The prepared materials were passivated in an argon-filled glovebox, where oxygen was present at a low partial pressure. The passivation lasted 48 h. Passivated materials were covered by hexane and stored in the laboratory for the duration of this project.

Reactivity was initially assessed by taking 100–200 mg of powder on a ventilated filter paper and igniting the paper by a butane lighter inside a fume hood. The 30 wt.% silicon powders burned quickly with a bright noiseless flash; the 50 wt.% silicon powders were comparatively slower and generated particle streaks jetting from the burning powder bulk. For the 50 wt.% silicon compositions, despite a coarser scale of mixing and poor homogeneity, 1-stage 50Si·50BiF_3_ was visibly more reactive than two-stage milled 50Si·50BiF_3_. There was no apparent difference between composites with BiF_3_ and CoF_2_ serving as oxidizers.

### 2.2. Material Characterization

The surface morphology and homogeneity of mixing between components in the prepared powders was examined using a JSM-7900 field emission scanning electron microscope (SEM) by JEOL, Tokyo, Japan. The imaging was performed by back-scattered electrons to achieve compositional contrast. To identify material composition and impurities incorporated by milling, energy dispersive X-ray spectroscopy (EDX) was used. The prepared powders were also analyzed using X-ray diffraction (XRD) on a PANalytical Empyrean multipurpose research diffractometer by Malvern Panalytical, Malvern, UK, operated at 45 kV and 40 mA using filtered Cu K_α_ radiation.

Reactivity of powders containing 50 wt.% silicon was tested by thermogravimetric analysis (TG) coupled with differential thermal analysis (DTA)/differential scanning calorimetry (DSC) on a STA409PG by Netzsch, Selb, Germany. The powders with a composition of 30 wt.% silicon were not tested to avoid releasing large amounts of aggressive fluorinated gases in the instrument.

Samples were heated in open alumina DTA crucibles only up to 973 K (700 °C) to limit release of volatile fluorinated species. Samples were tested in argon (99.998%) flowing at 50 mL/min as an inert environment and in an oxidizing environment, with an additional 50 mL/min flow of oxygen (99.994%). Both gases were sourced from Airgas. For each case, a baseline was obtained by heating an empty crucible under identical conditions. The sample mass was fixed at 5 mg, except for a few runs in argon performed with larger sample masses of 10 and 24 mg. For the oxidizing environment, heating rates 1, 2 and 5 K/min were used. Experiments in argon were performed with heating rates of 2, 5 and 10 K/min. To probe the progress of reactions, samples were recovered from specific intermediate temperatures and analyzed using XRD.

### 2.3. Heated Filament Ignition

The ignition temperatures for the prepared powders were obtained by coating a sample onto an electrically heated 24 gauge (0.5105 mm) nickel-chromium wire. The time of ignition was determined from a high-speed video and the wire temperature was measured optically, using a calibrated photo diode. The basic experimental setup and procedure have been presented earlier [27]. The photodiode-based pyrometer has been described in Ref. [24]. Briefly, the wire was coated with a suspension of the powder in hexane. Once the hexane dried, the wire was electrically heated using a variable set of rechargeable large cell batteries (7238K57 McMaster Carr. Elmhurst, IL, USA) and a 1 Ω rheostat connected in series to achieve targeted heating rates in the range of 10^3^–10^4^ K/s. A fiber optics cable was focused at a section of the wire that was uncoated and fed into germanium photodiode (PDA30B2 by Thorlabs, Newton, NJ, USA). The photodiode was calibrated against a black body emission source (BB4A by Omega Engineering, Bridgeport, NJ, USA) to function as a high-speed pyrometer. To correlate the time of ignition to the temperature of the wire, the ignition was recorded using a MotionPro 500 camera by Redlake, San Diego, CA, USA, synchronized with the ignition circuit. At least 5 runs were performed for each targeted heating rate.

### 2.4. Particle Combustion

The particle combustion was studied by aerosolizing the materials in a room-temperature air stream fed into the focal spot of a sealed CO_2_ laser (Evolution 125 by Synrad, Mukilteo, WA, USA). The optical emission of the ignited particles was captured by filtered photomultiplier tubes (PMT) to obtain burn times. Each burning particle produced an emission pulse; the pulse width taken at 10% of its peak value was interpreted as the burn time.

The powder feeder described elsewhere [28] consisted of a screw driven by a DC motor, with 30 threads coated by 0.12 g of powder. The powder lodged between the threads was blown off the screw and aerosolized by a carrier gas, air flown at 0.68 L/min. The aerosolized particles were fed via a 2.39 mm internal diameter brass tube. The upper end of the tube was placed 2 mm below the focal spot of the laser. The laser beam was focused by a ZnS lens to a spot of about 250 μm diameter. The laser was operated at 37.5 W, or ca. 30% of its maximum power, which is much higher than the ignition threshold for the materials tested. Thus, particles passing through the laser beam ignited consistently. Further experimental details are available elsewhere [29].

A fiber optics bundle trained to capture the particle emissions was placed 2 cm above and 16 cm away from the brass feeder tube. The fiber was split to feed to two R3896-03 PMTs by Hamamatsu, Hamamatsu City, Japan, filtered at 700 and 800 nm. The signal from the PMTs was acquired through a 16 bit PCI-6123 board by National Instruments, Austin, TX, USA, at 100,000 samples per second and processed using LabView software (Labview 16 by National Instruments, August 2016). Signal was collected for periods of 8 s at a time. Several such 8 s runs were required to acquire over 800 pulses produced by individual burning particles for each material. Overlapping, or closely spaced pulses were dismissed. The widths of the collected individual particle pulses, interpreted as burn times, were developed into respective statistical distributions employing a smoothing kernel density function [21].

### 2.5. Aerosolized Particle Collection and Sizing

The aerosolization of the composite powders may cause size classification. Thus, the size distributions for the particles observed to burn in the present experiments could differ from those of the prepared powders. To account for this, samples of powders passed through the feeder were collected and analyzed. A flat aluminum substrate covered with a double-sided adhesive carbon tape was placed 2.5 cm above the brass feeder. Particles were trapped onto the tape for 10–15 s using the feeder operated at the same gas flowrate as in the combustion tests; however, with the laser beam turned off. The collected particles were imaged by the SEM and their sizes were obtained using ImageJ software [30]. From a table of all particles of one material, size distributions were generated using a kernel density function. The particle size distributions were correlated with the burn time distributions to identify the effect of particle size on the burn time. Such correlations broadly assume that larger particles burn longer.

## 3. Results

### 3.1. Particle Morphology and Composition

**Figure 1:**
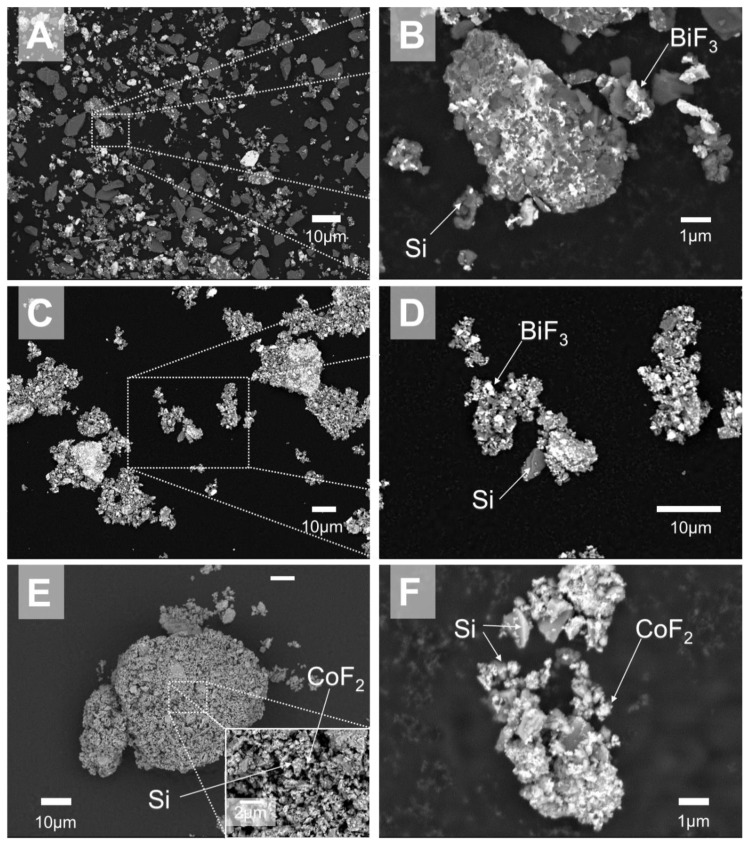
Backscattered electron images of as-milled 50 wt.% silicon-50 wt.% fluoride composites: (**A**,**B**) 1-stage 50Si·50BiF_3_, (**C**,**D**) 50Si·50BiF_3_ and (**E**,**F**) 50Si·50CoF_2_.

SEM images of the prepared 50 wt.% silicon- 50 wt.% metal fluoride composites are presented in Figure 1. The grey crystalline phase was silicon, and the brightest phase was the fluoride. The carbon tape in the background appeared darkest. The single stage milled 1-stage 50Si·50BiF_3_ powder is shown in Figure 1A,B. It is apparent from Figure 1A that the fluoride was not uniformly distributed in the material. Many relatively large, ca. 20–40 μm, Si particles and some unattached BiF_3_ clusters were observed. Part of the image in Figure 1A with a relatively well-homogenized composite particle was magnified and shown as Figure 1B. Individual Si and BiF_3_ phases were interspersed on a submicron scale. Such particles were few with a majority of silicon particles having little to no fluoride attached to them.

A composite prepared using the two-stage protocol, 50Si·50BiF_3_ is shown in Figure 1C,D. All particles show good mixing of the fluoride and silicon and reduced particle sizes in Figure 1C. A magnified image of a representative particle in Figure 1D shows angular silicon crystals decorated by bright particles of BiF_3_.

The images of the two-stage milled 50Si·50CoF_2_ powder are presented in Figure 1E,F. In Figure 1E, a large, agglomerated particle of 60 μm, with no discernable difference in phase contrast is seen. Such agglomerates were common. The inset in Figure 1E shows that the particle surface had distinct angular platelets and smaller particles. Through EDX, the larger platelets were confirmed to be Si and the smaller particles were found to be CoF_2_. A smaller particle of 50Si·50CoF_2_ is shown in Figure 1F. Distinct morphology enables one to identify both silicon and CoF_2_ phases mixed rather uniformly on the nanoscale.

**Figure 2:**
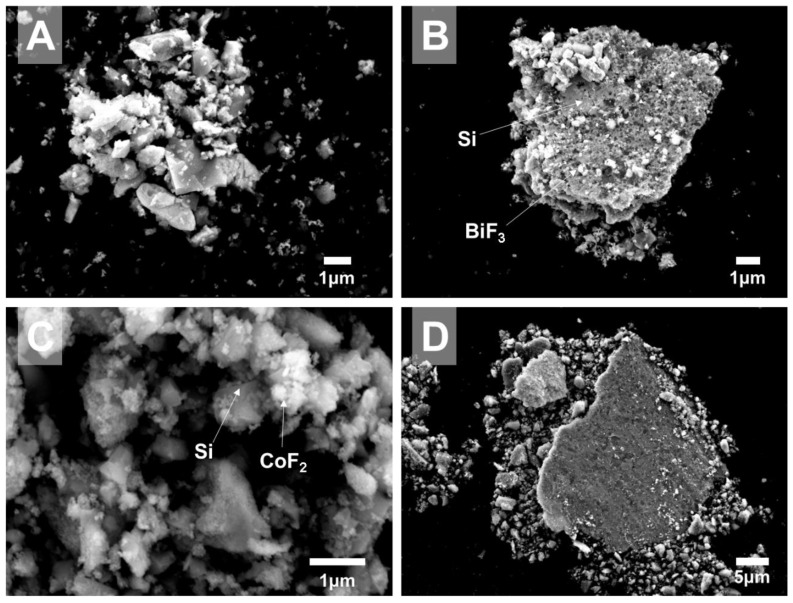
Secondary electron images of as milled 30 wt.% silicon-70 wt.% fluoride composites: (**A**,**B**) 30Si·70BiF_3_ and (**C**,**D**) 30Si·70CoF_2_.

SEM images of powders with 30 wt.% silicon and 70 wt.% metal fluoride are presented in Figure 2. These images were obtained using secondary electrons to highlight the surface morphology, while losing on the phase contrast. General particle morphology for 30Si·70BiF_3_ is illustrated in Figure 2A,B. Similarly, the morphology for 30Si·70CoF_2_ is illustrated by Figure 2C,D.

A large amount of unattached BiF_3_ particles and small crystalline silicon particles were observed in Figure 2A. Some relatively large BiF_3_ particles decorated agglomerates of silicon crystals (Figure 2B); although the coating did not appear to be uniform.

In Figure 2C, cobalt fluoride was observed as small fuzzy particles covering jagged crystals of silicon. No unattached CoF_2_ was observed. Generally, 30Si·70CoF_2_ composite contained silicon particles with a broad size distribution and all Si surfaces were rather uniformly coated by cobalt fluoride, unlike 30Si·70BiF_3_. Occasional large particles (>10 μm) were observed in both materials, as shown in Figure 2B,D. A flat surface, typically formed by milling balls pressing into a softer material, was commonly observed for such particles. SEM images show that such large particles consisted of fine silicon particles embedded in a fluoride matrix.

**Figure 3:**
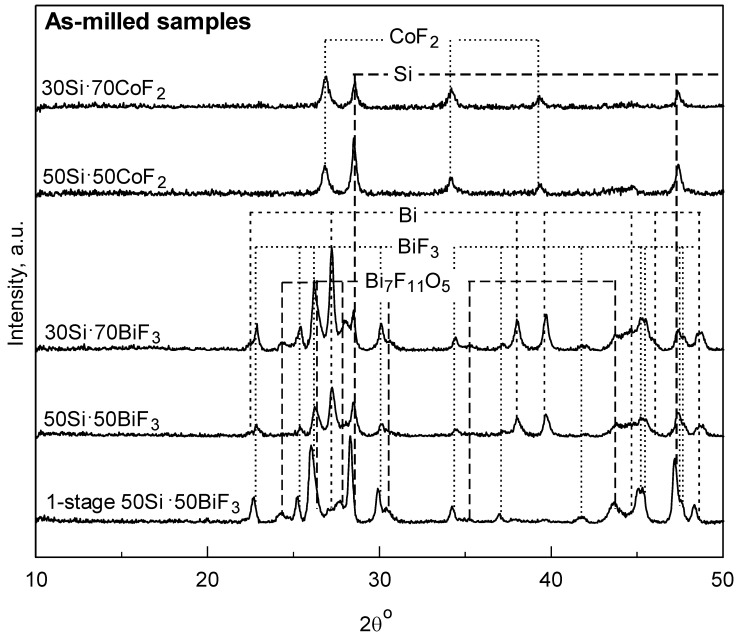
XRD patterns of the prepared composite powders.

The XRD patterns of milled composites are presented in Figure 3. Peaks corresponding to BiF_3_ and Bi_7_F_11_O_5_ [31] were observed in all Bi-containing composites. Detectable amounts of reduced Bi were also observed. In the two-stage milled composites, the fluoride and reduced Bi peak intensities scale with the loaded BiF_3_ concentration; stronger fluoride peaks were observed for 30Si·70BiF_3_ as compared to 50Si·50BiF_3_. Results of quantitative composition analysis for BiF_3_-containing composites based on the whole pattern refinement performed using the X-pert Highscore software package [32] are shown in Table 3. The data suggest that 50Si·50BiF_3_ retained only ca. 64% of the initial fluorine. On the other hand, the poorly refined 1-stage 50Si·50BiF_3_ exhibited the weakest bismuth peaks and strongest BiF_3/_Bi_7_F_11_O_5_ peaks among the three Si·BiF_3_ composites; it retained approximately 71% if the initial fluorine.

The XRD pattern of the composites with CoF_2_ shows peaks corresponding to silicon and cobalt fluoride. No reduced cobalt is observed. The scaling of the fluoride peak intensity as a function of the loaded fluoride concentration was less apparent.

### 3.2. Thermal Analysis

**Figure 4:**
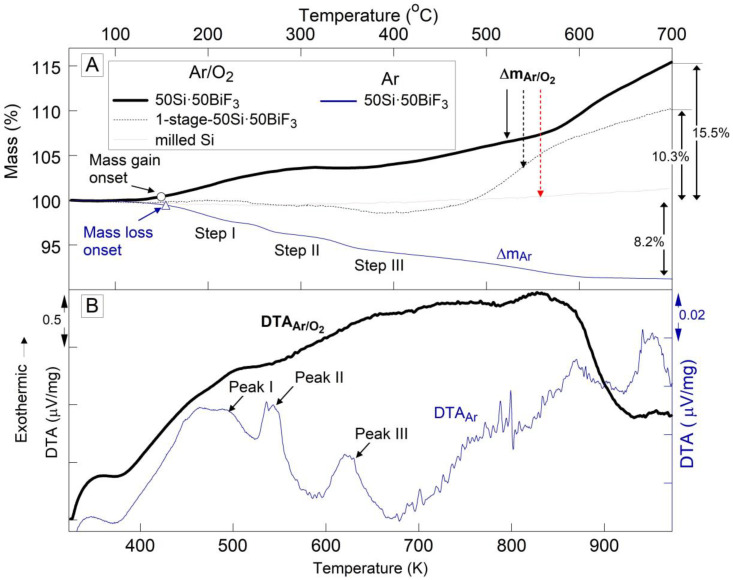
(**A**) Thermogravimetric analysis (TG) and corresponding (**B**) differential thermal analysis (DTA) traces for 50Si·50CoF_2_ heated at 5 K/min in different environments.

The TG and their corresponding DTA traces for 50Si·50CoF_2_ heated in both oxidizing and inert environments are shown in Figure 4. The DTA traces for the oxidizing and inert runs used separate vertical scales because of difference in the heat effects observed. The TG trace measured in Ar (Figure 4A) shows a single mass loss event with the onset below 673 K (400 °C); the total observed mass loss was close to 23% of the initial mass. The respective DTA trace (Figure 4B) shows weak features, which can be interpreted at two broad exothermic humps (peaks at 498 and 893 K (or 225 and 620 °C)) or a very broad exothermic feature overlaid with an endothermic event. To support the latter interpretation, much of the endothermic feature correlated with the observed mass loss.

For TG in Ar/O_2_, the onset for a gradual mass gain was at 548 K (275 °C). The mass gain was reversed at 723 K (450 °C), and the maximum mass loss of ca. 8.3% was observed by 798 K (525 °C). After the minimum, the mass began increasing again. The corresponding DTA trace exhibited a rising baseline (despite the correction applied using an empty crucible). A small exothermic peak was noted at 280 °C, corresponding to the onset of the mass gain. A stronger exotherm was observed at 773 K (500 °C) correlating with the mass loss.

**Figure 5:**
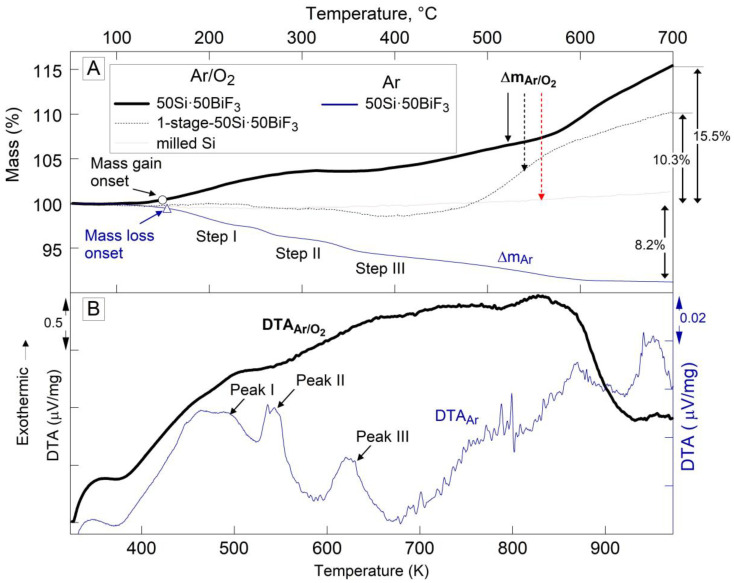
(**A**) TG and corresponding (**B**) DTA traces for 50Si·50BiF_3_ in heated at 5 K/min in oxidizing and inert environments. The oxidative TG runs for 1-stage 50Si·50BiF_3_ and milled Si are also shown.

The TG and DTA plots for 50Si·50BiF_3_ heated in different environments are presented in Figure 5. In Figure 5A, the oxidative TG runs for the milled/size-reduced Si and 1-stage 50Si·50BiF_3_ were presented for comparison. No mass change is observed for the Si reference.

The TG trace for 50Si·50BiF_3_ in Ar shows a gradual mass loss of 8.2%. It begins around 423 K (150 °C) and ends by 873 K (600 °C). Weak but reproducible step-like features are noted in the TG trace; the onset for the mass loss are marked by a filled circle as labeled in Figure 5A. The corresponding DTA plot in Figure 5B, shows multiple weak peaks. It is possible to interpret the trace as showing a broad endothermic peak, similar to that in Figure 4. Additionally, several exothermic features are noted. The first three of these features, labeled in Figure 5B, correlated with the mass loss steps. The second exotherm, at ca. 543 K (270 °C; marked Peak II), had a small but reproducible superimposed endothermic feature.

For 50Si·50BiF_3_ experiments in both Ar and Ar/O_2_ were performed at different heating rates. The shift of the respective mass loss and mass gain points to higher temperatures at greater heating rates was used to consider relevance of the respective reactions to ignition, as discussed below.

In Ar/O_2_, 1-stage milled 50Si·50BiF_3_, exhibited a small mass loss (ca. 1.5%, onset at 583 K (310 °C)) and then mass gain, which was not complete by the end of the run. For the two-stage milled composite, 50Si·50BiF_3_, heated in Ar/O_2_, the mass began to increase at ca. 423 K (150 °C) as marked in Figure 5A. The mass was stabilized by about 593 K (320 °C). It began increasing again at 658 K (385 °C); the rate of mass gain increased around 873 K (600 °C). The final measured mass gain was 15.5%, it was not complete by the end of the run. The corresponding DTA curve for 50Si·50BiF_3_ seen in Figure 5B exhibited a rising baseline indicating a broad exothermic process initiated at least from 393 K (120 °C). The exothermic feature ended around 923 K (650 °C).

For both oxidizing and inert environments runs for 50Si·50BiF_3_, partially reacted samples were recovered from 473, 673 and 973 K (200, 400 and 700 °C).

**Figure 6:**
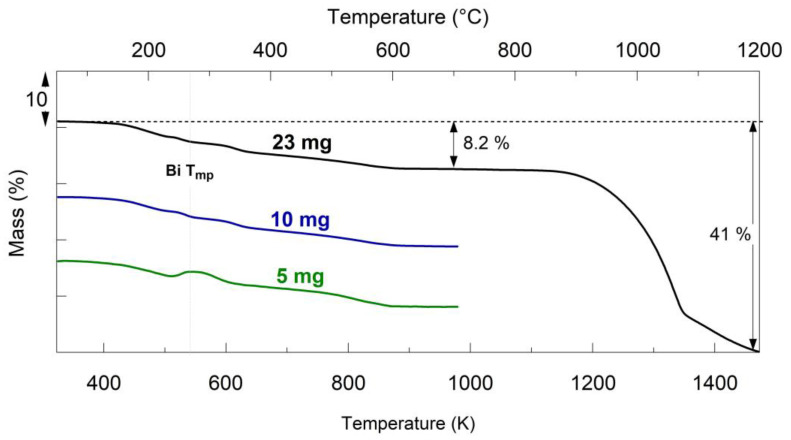
TG traces for different mass samples of 50Si·50BiF_3_ heated in Ar at 5 K/min.

To better resolve weak processes occurring in Ar, runs with the powder mass increasing from 5 to 10 and to 23 mg were performed for 50Si·50BiF_3_. The respective TG traces are presented in Figure 6. The step-like features observed during mass loss were prominent for the lowest sample loading of 5 mg, for which a mass gain was also noted around 543 K (270 °C), which was close to the melting temperature of bismuth (marked in the Figure 6). The mass gain could have been caused by oxidation of the sample reacting with trace oxygen remaining in the furnace despite it being flushed with Ar. Melting of Bi could have accelerated such oxidation. When the sample was heated to a higher temperature of 1473 K (1200 °C), cf. 23 mg trace, a strong mass loss of 33% was observed with an onset at 1173 K (900 °C), which could only be associated with the loss of Bi.

**Figure 7:**
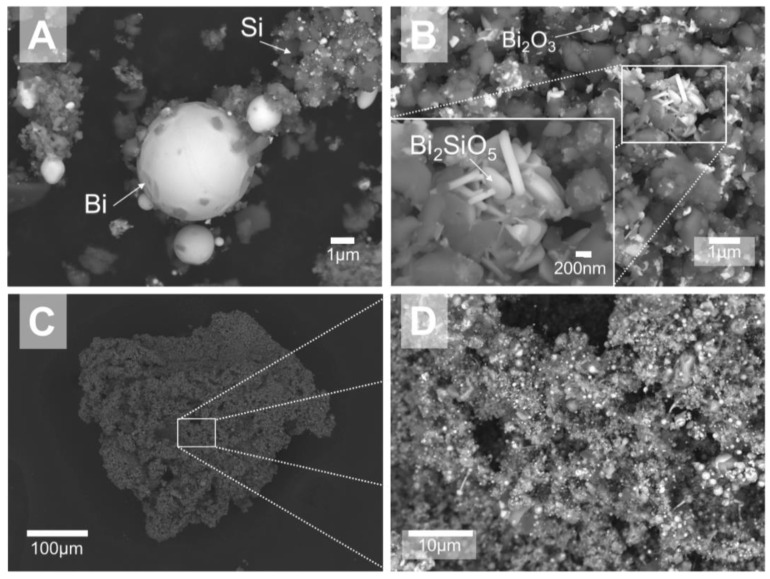
Backscattered electron images of the two-stage milled composite 50Si·50BiF_3_ heated at 5 K/min and recovered from 973 K (700 °C): (**A**) in Ar, (**B**) in Ar/O_2_ and recovered from 1473 K (1200 °C) in Ar (**C**,**D**).

SEM images of partially reacted 50Si·50BiF_3_ recovered from different temperatures in inert and oxidizing environments are presented in Figure 7. The powder heated to 700 °C in Ar (Figure 7A) shows bright spherical bismuth particles studded on darker silicon particles. The dispersion of bismuth across silicon particles was largely preserved. For the sample heated to 700 °C in Ar/O_2_ (Figure 7B), elongated crystals, appearing to be Bi_2_SiO_5_ from EDX analysis, are observed; see the inset for details. Minor bright spheroidal particles rich in Bi were found decorating the surface of the darker Si-rich matrix. EDX shows oxygen present in all phases.

Figure 7C shows a characteristic particle heated to 1473 K (1200 °C) in Ar. It is porous and consists of several fused parts. Figure 7D provides a higher magnification image, presenting the surface morphology with multiple small spherical submicron bismuth-rich particles and lamellae of silicon crystal. In materials heated in both Ar and Ar/O_2_, EDX detects iron and chromium; both contaminants came from the steel milling balls.

**Figure 8:**
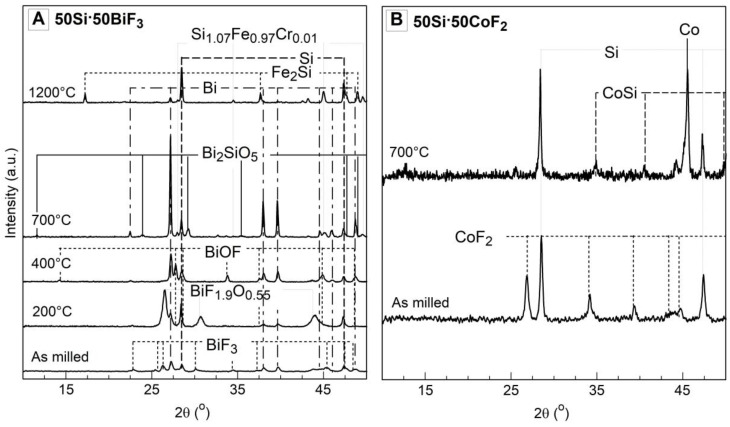
The XRD traces of the two-stage milled composites: (**A**) 50Si·50BiF_3_ and (**B**) 50Si·50CoF_2_ heated at 5 K/min in Ar and quenched at different temperatures.

XRD patterns for 50Si·50BiF_3_ and 50Si·50CoF_2_ heated to different temperatures in Ar and recovered for analysis are presented in Figure 8, along with the reference pattern for the as-milled material. The specific peaks of oxyfluoride Bi_7_F_11_O_5_ in the as milled sample have not been presented in the Figure 8 due to space constraints.

Despite Ar purge, traces of oxygen were expected in these runs. For 50Si·50BiF_3_ (Figure 8A) peaks associated to BiF_3_ are depressed by 473 K (200 °C). Simultaneously, BiF_1.9_O_0.55_ peaks [33] became dominant and Bi_7_F_11_O_5_ peaks from starting powder disappeared. At 673 K (400 °C), strong peaks of Bi, Si and the fluorine containing BiOF became predominant. By 973 K (700 °C), peaks corresponding to fluorinated species disappeared, remaining were peaks of Bi and Si and minor peaks attributed to Bi_2_SiO_5_ and Fe_2_Si. At 1473 K (1200 °C), Bi peaks diminished, and peaks of Si became stronger. Patterns attributed to iron and chromium impurities, such as Fe_2_Si and Si_1.07_Fe_0.97_Cr_0.01_ were also noted.

For 50Si·50CoF_2_, the XRD patterns of the starting powder and of the material heated to 700 °C in Ar are shown in Figure 8B. CoF_2_ peaks disappeared for the heated material; aside from elemental Co and Si, a pattern for CoSi was observed.

**Figure 9:**
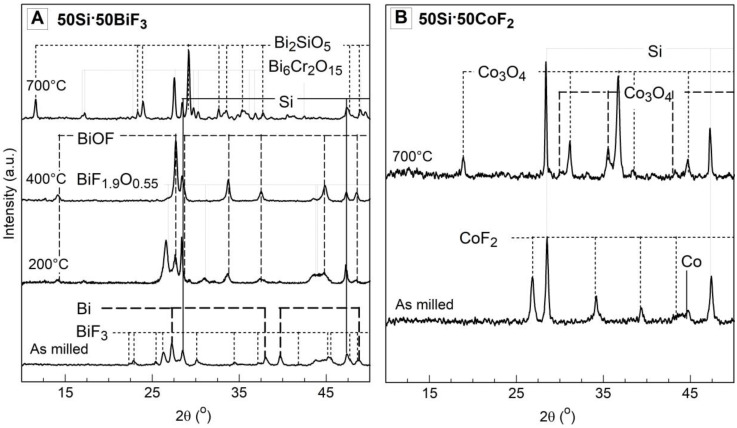
XRD patterns of the two-stage milled composites (**A**) 50Si·50BiF_3_ and (**B**) 50Si·50CoF_2_ heated at 5 K/min in Ar/O_2_ and recovered from different temperatures.

XRD patterns for the powders heated to different temperatures in Ar/O_2_ for 50Si·50BiF_3_ and 50Si·50CoF_2_ are presented in Figure 9. As seen in Figure 9A, the as milled 50Si·50BiF_3_ had prominent peaks of BiF_3_, Si, Bi and weaker peaks of Bi_7_F_11_O_5_. The material recovered from 473 K (200 °C) exhibited weaker BiF_3_ peaks while Bi and the oxyfluoride BiF_1.9_O_0.55_ peaks became stronger. By 673 K (400 °C), the BiF_3_ peaks were absent, Si peaks diminished appreciably, while a new oxyfluoride species, BiOF was observed. By 973 K (700 °C), oxidized products, Bi_2_SiO_5_ and Bi_6_Cr_2_O_15_ were the primary species. Minor peaks corresponding to Si were observed as well.

The XRD patterns for as-milled 50Si·50CoF_2_ and that quenched at 973 K (700 °C) in Ar/O_2_ are presented in Figure 9B. Strong peaks of the oxide of cobalt, Co_3_O_4_ and unreacted Si were observed.

### 3.3. Heated Filament Ignition

**Figure 10:**
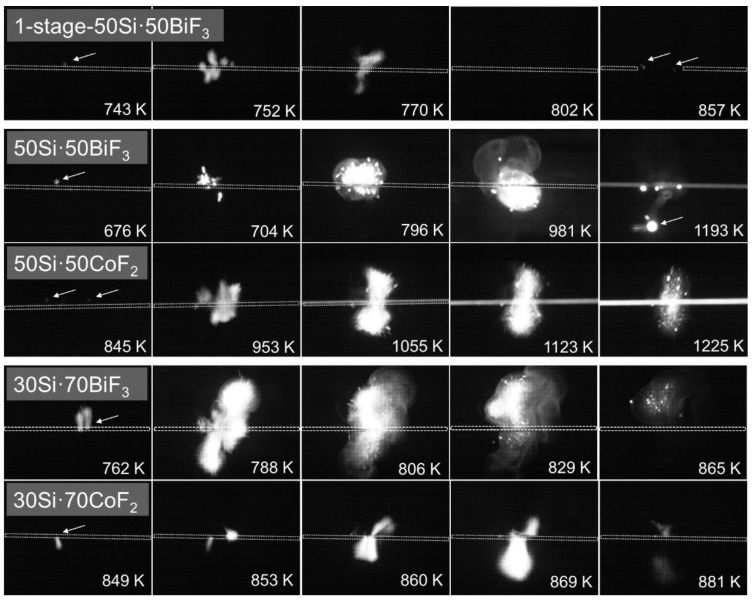
High-speed video frames of the ignition of 1-stage 50Si·50BiF_3_ (heated at 2100 K/s) and two-stage milled materials; 50Si·50BiF_3_ (heated at 3800 K/s), 50Si·50CoF_2_ (heated at 2100 K/s), 30Si·70BiF_3_ (heated at 2200 K/s) and 30Si·70CoF_2_ (heated at 2200 K/s), on an electrically heated nichrome wire. The wire temperatures are indicated.

High-speed video frames for all materials igniting on a heated filament are presented in Figure 10. The frames are labeled with the temperature of the filament.

All prepared materials exhibited ignition at low temperatures well before the filament turns incandescent. The incandescence of particles marks the ignition in fuel-rich 50 wt.% silicon compositions as indicated by arrows in the respective first frames. The 1-stage 50Si·50BiF_3_ composite exhibited less bright, short-lived particle streaks within an incandescent plume. At higher temperatures (see 857 K) a weak secondary ignition event was observed as marked in the frame.

For 50Si·50BiF_3,_ bright smoke enveloped the burning particles flying off the filament, as seen in frames captured above 796 K. The 50Si·50CoF_2_ material ignited producing a bright plume with few particle streaks, which became prominent at higher wire temperatures (>1123 K).

For materials with higher fluoride content, 30Si·70BiF_3_ and 30Si·70CoF_2_, ignition caused luminescent plume flares. For 30Si·70BiF_3_, particle streaks and smoke were observable above 806 K. The 30Si·70CoF_2_ composite generated a bright plume and no detectable burning particles.

**Figure 11:**
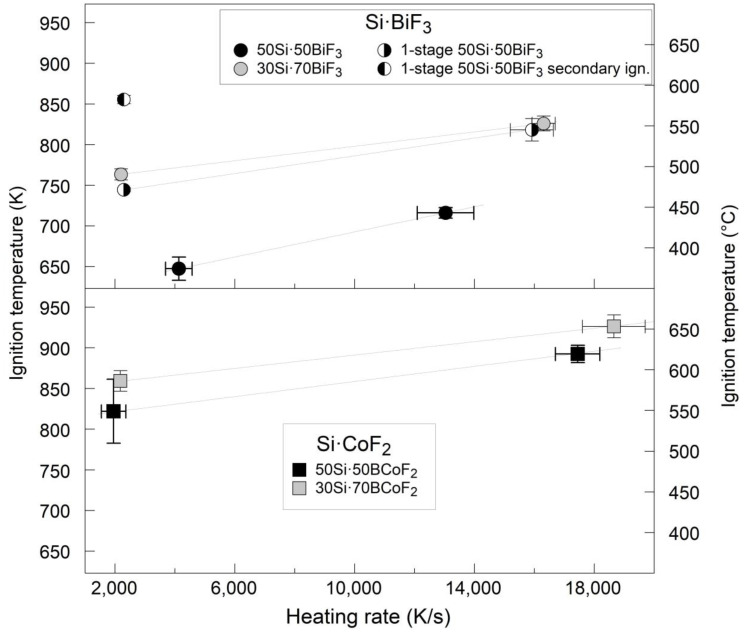
Ignition temperature in air of all prepared materials as a function of the heating rate.

The measured ignition temperatures of all prepared materials are shown as a function of heating rates in Figure 11. The error bars represent the scatter in 5 runs performed for each targeted heating rate. All ignition temperatures increase with the heating rate.

Lower ignition temperatures were measured for the materials with BiF_3_ compared to those with CoF_2_. The composites with 50 wt.% Si ignited consistently at lower temperatures compared to the composites with 30 wt.% Si. The 1-stage 50Si·50BiF_3_ had a higher ignition temperature comparable to its two-stage milled analog, 50Si·50BiF_3_. The secondary ignition for 1-stage 50Si·50BiF_3_ could only be clearly detected at lower heating rates. At higher heating rates, this secondary ignition could have occurred at much higher temperatures and was not discernable due to the bright filament emission.

### 3.4. Particle Combustion

In experiments employing the laser ignition of composite particles, 50Si·50CoF_2_ powder could not be fed consistently. Conversely, 50Si·50BiF_3_ powder was easy to feed; thus, only the experimental results with this powder are presented. Attempts to ignite milled Si powder, which could be fed into the laser beam, were not successful. Additionally, condensed combustion products could not be collected for analysis, likely because of the substantial amounts of gaseous combustion products.

**Figure 12:**
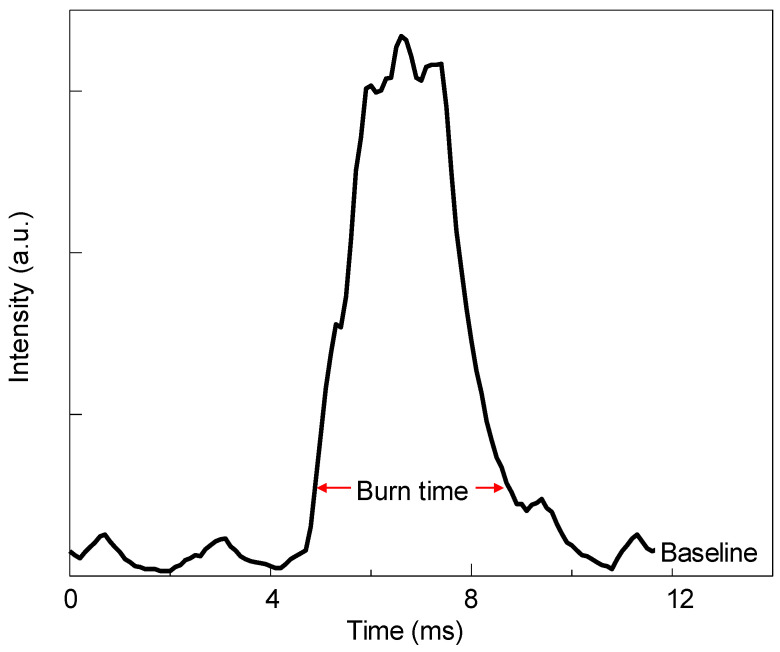
A representative emission pulse produced by a 50Si·50BiF_3_ particle burning in air.

**Figure 13:**
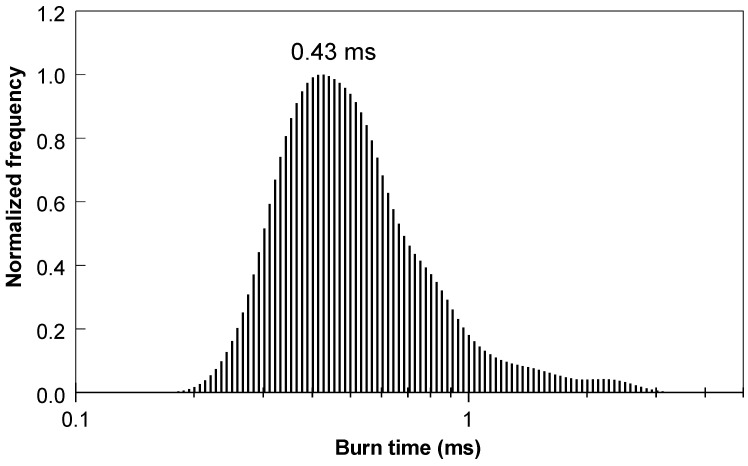
The burn time distribution for particles of 50Si·50BiF_3_ burning in air.

A representative emission pulse of a laser-ignited 50Si·50BiF_3_ particle burning in air is shown in Figure 12. All pulses observed had single peaks with minor oscillatory features. A distribution of the particle burn times is shown in Figure 13. It peaked at 0.43 ms with a tail trailing towards longer times.

**Figure 14:**
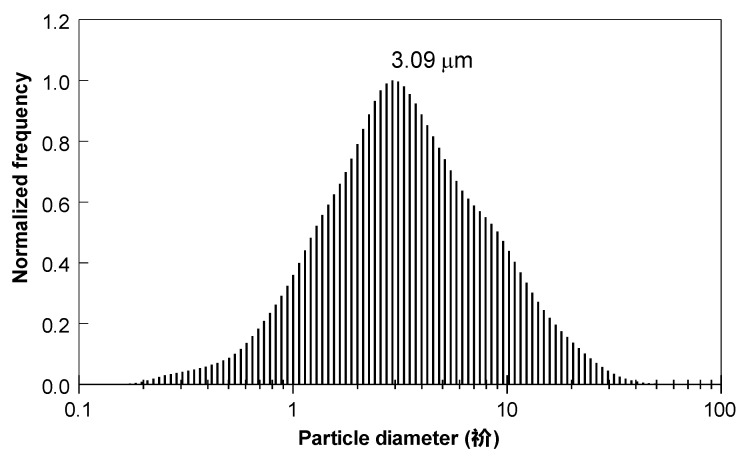
The particle size distribution for aerosolized 50Si·50BiF_3_ powder passed through the feeder.

The size distribution of the aerosolized 50Si·50BiF_3_ particles passed through the feeder is shown in Figure 14. The distribution was nearly symmetrical, with a mode of 3.09 µm.

**Figure 15:**
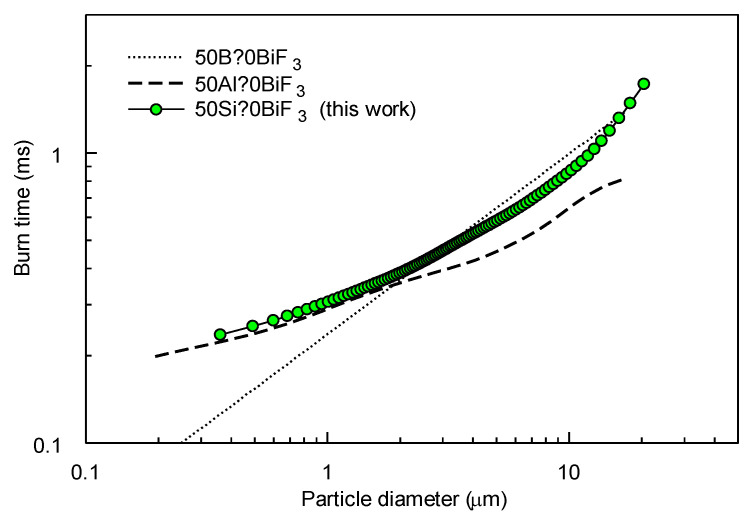
Burn time–particle size correlation for 50Si·50BiF_3_ powder ignited in air by a laser. For comparison, similar correlations are shown for powders of 50Al·50BiF_3_ [20] and 50B·50BiF_3_ [23] obtained by arrested reactive milling.

The burn time distribution was correlated with the particle size distribution for 50Si·50BiF_3_; the correlation plot is presented in Figure 15 along with earlier results for other metal-metal fluoride composites obtained using the same data processing method [20,23]. Like Al and B-based composites using BiF_3_ as an oxidizer, 50Si·50BiF_3_ composite powder particles burn rapidly, with most burn times under 1 ms. These times were shorter than reported burn times for Al particles of the same sizes.

The adiabatic flame temperature and predicted mole fractions of the combustion products of 50Si·50CoF_2_ burning in air calculated by NASA CEA code [34] are presented in the Supplementary Section Figure S1. The particle surface temperature during combustion is expected to be close to 2750 K (predicted to be the adiabatic flame temperature), sufficiently high for gasifying a significant fraction of the main combustion product SiO_2_.

## 4. Discussion

### 4.1. Material Preparation

A single-stage milling protocol does not effectively mix the physically different fluoride and silicon powders. Conversely, using premilled silicon for the second step milling with metal fluorides yields well-homogenized nanocomposite powders.

Materials with CoF_2_ have distinct morphological features, where the fluoride formed a coating on the larger silicon particles. Even with increased fluoride content, no loose fluoride or uncoated silicon particles were observed in 30Si·70CoF_2_. It is surprising that such a fine scale of mixing was achieved without detectable mechanochemical reaction, e.g., lack of reduced Co detected by XRD of as-milled powders (see Figure 3). The XRD results were consistent with the thermal analysis, Figure 4A. The complete reduction of CoF_2_ by silicon to form gaseous SiF_4_ resulted in a mass loss of 26%. This was close to the mass loss of 23 wt.% measured by TG.

Based on XRD of the as-milled 50Si·50BiF_3_ showing the presence of reduced bismuth and bismuth oxyfluorides, it was estimated that BiF_3_ made up only 28.5–30 wt.%, with the remaining 20–21.5 wt.% of BiF_3_ lost before or during milling. Accounting for this initial amount of BiF_3_ available in the as-milled powder, it is expected that the complete reaction of Si with the remaining F in the material would result in an observable mass loss of close to 8.9%. This agreed reasonably with the mass loss of 8.3% observed in TG (Figure 7A). While a smaller fraction of the fluoride reacted and was lost for the 1-stage 50Si·50BiF_3_, the mixing scale and homogeneity for that composite material were inadequate.

### 4.2. Low-Temperature Reactions

The TG for 50Si·50BiF_3_ and 50Si·50CoF_2_ in Ar shows a mass loss due to the evolution of SiF_4_, described by reaction (1). As seen in Figure 4, SiF_4_ formation in 50Si·50CoF_2_ occurred in a single step between 661 and 873 K (388 and 600 °C). Conversely, for 50Si·50BiF_3_, the mass decreased gradually, with discernable stages starting from ca. 423 K (150 °C) and extending to 873 K (600 °C).

In Ar/O_2_ oxidation of the reduced metal and oxidation of silicon occur parallel to fluorination of silicon. Both oxide formation reactions form condensed products and thus increase the mass. Assuming for simplicity that the fluorination occurs similarly in both Ar and Ar/O_2_ environments, the mass gain due to oxidation, $\Delta m_{ox}$, can be estimated from the mass difference between measurements in Ar/O_2_ and in Ar:

(2) $$\Delta m_{ox}=\Delta m_{Ar/O_{2}}-\Delta m_{Ar}$$

**Figure 16:**
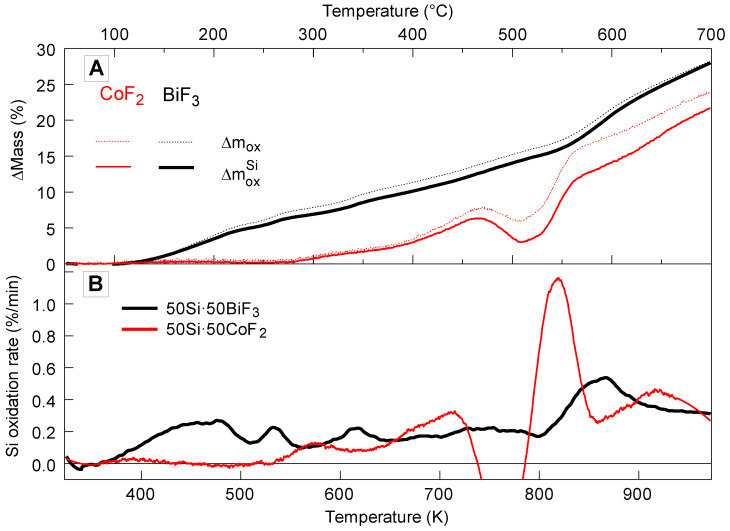
The (**A**) oxidative mass gain and corresponding (**B**) silicon oxidation rate of two-stage milled materials 50Si·50BiF_3_ and 50Si·50CoF_2_.

This change of mass is shown in Figure 16. The mass gain due to oxidation consists of two components, caused by oxidation of silicon, $\Delta m_{ox}^{Si}$ and that of the reduced metal, $\Delta m_{ox}^{Me}$. The value of $\Delta m_{ox}^{Me}$ can be estimated from the mass measured in Ar, assuming $\Delta m_{Ar}$ is entirely due to evolution of SiF_4_ and assuming that all the reduced metal oxidizes immediately when the reaction occurs in Ar/O_2_:

(3) $$\Delta m_{ox}^{Me}=\Delta m_{Ar}^{Me}=\frac{M_{Me}}{M_{SiF_{4}}}\cdot \frac{x}{4}\Delta m_{Ar}$$

Subtracting this mass from $\Delta m_{ox}$ gives:

(4) $$\Delta m_{ox}^{Si}=\Delta m_{ox}-\Delta m_{ox}^{Me}$$

Finally, the rate of Si oxidation can be obtained dividing the derivative $d\left(\Delta m_{ox}^{Si}\right)/dt$ by the mass of Si remaining in the material accounting for SiF_4_ leaving as gas (Equation (1)), also based on the TG measured in Ar.

Processing experimental traces using Equations (2) and (4) yields traces for $\Delta m_{ox},\;\Delta m_{ox}^{Si}$, and the rate,$\;\frac{1}{m_{Si}}\frac{d\left(\Delta m_{ox}^{Si}\right)}{dt}$ shown in Figure 16. Figure 16B shows that the rate of silicon oxidation parallels the observed rate of oxidation in the Ar/O_2_ environment. Since only as much Me oxide can form as MeF_x_ has been reduced, the bulk of the observed mass gain represents the oxidation of Si. The apparent negative Si oxidation rate seen for 50Si·50CoF_2_ at between 450 and 500 °C suggests that the assumption that SiF_4_ formation is identical in Ar and Ar/O_2_ environments is not strictly true.

For 50Si·50CoF_2_, Figure 16 suggests that oxidation of Si began at a higher temperature than the corresponding SiF_4_ formation (cf. Figure 4); it also began at a higher temperature than for 50Si·50BiF_3_, for which oxidation began simultaneously with fluorination (cf. Figure 5). For both composites, the oxidation rate of Si increased initially and then became relatively stable over a range of temperatures. For both composites the low-temperature rates of heat release associated with the observed initial rate of oxidation of Si could be added to the rates of heat release caused by the Si fluorination occurring simultaneously (e.g., recovered from data in Figure 4 and Figure 5 for 50Si·50CoF_2_ and 50Si·50BiF_3_, respectively) to describe the exothermic reactions leading to ignition of such composite materials.

Aside of relatively small changes in the reaction rate for 50Si·50BiF_3_, a stepwise increase in the oxidation rate is observed between 803 and 973 K (530 and 600 °C). As discussed below, this accelerated oxidation at relatively high temperatures was unlikely to affect ignition of the prepared composite. For 50Si·50CoF_2_ the apparent oxidation rate of Si dropped around 723 K (450 °C). However, based on Figure 5, it can be concluded that this effect was superficial and simply means that the fluorination of Si accelerated rapidly at such temperatures, with the associated mass loss becoming stronger than mass gain due to oxidation of both Si and reduced Co. Indeed, after a while, the oxidation rate of Si returned to about the same level it was at just below 723 K.

### 4.3. Reactions Leading to Ignition

**Figure 17:**
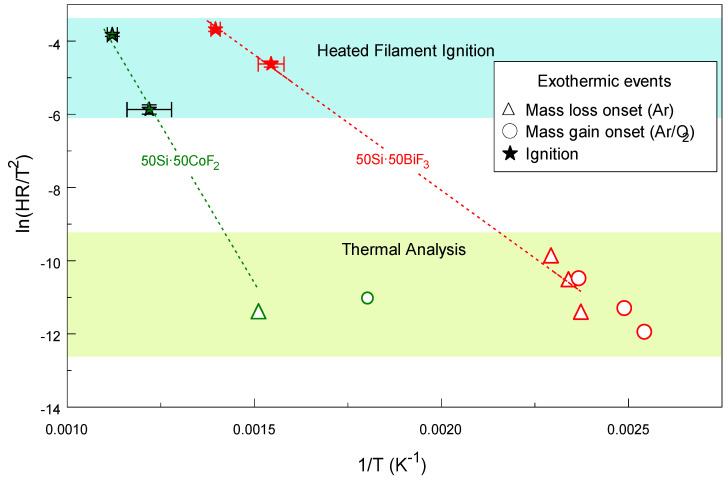
Kissinger plot combining TG and DTA features and heated filament ignition data for two-stage milled 50Si·50BiF_3_ and 50Si·50CoF_2_.

The onsets of TG features observed in both inert and oxidizing environment runs for both 50Si·50BiF_3_ and 50Si·50CoF_2_ were plotted along with their respective heated filament ignition temperatures in the Kissinger plot shown in Figure 17. The onsets of the mass loss observed in Ar and mass gain in Ar/O_2_ for 50Si·50BiF_3_ nearly coincided with each other for different heating rates. Extrapolating the kinetic trend from the ignition data points down to the range of low heating rates used in thermal analysis pointed clearly to the onsets of the observed mass gain (in Ar/O_2_) and loss (in Ar) as reactions associated with ignition. As discussed above, for 50Si·50BiF_3_, both fluorination and oxidation of Si occurred nearly simultaneously with both exothermic reactions contributing to igniting the material.

The onset of mass loss observed in Ar for 50Si·50CoF_2_ (for which the measurement was only made at one heating rate) was shifted from that of the mass gain observed for this material heated in Ar/O_2_ at a lower temperature. The kinetic trend implied by the respective ignition temperatures lined up better with the mass loss observed in Ar, and thus it was likely that the ignition was mostly associated with SiF_4_ formation for this composite. It is nonetheless expected that the low-temperature oxidation of Si, once started, assisted ignition. The apparent activation energy that can describe ignition was roughly evaluated from the slopes of line fits shown in Figure 17. These activation energies were around 62 and 140 kJ/mol for 50Si·50BiF_3_ and 50Si·50CoF_2_, respectively. For 50Si·50BiF_3_, the inferred activation energy was comparable to that reported for fluorination of silicon wafers by elemental fluorine at reduced fluorine partial pressures [35].

The higher temperature reactions are not shown but would be shifted far left in Figure 17 and thus would be clearly irrelevant for ignition.

### 4.4. Particle Combustion

The micron-sized silicon particles could not be initiated in the laser-assisted combustion experiment. Conversely, the prepared 50Si·50BiF_3_ composite could be readily initiated and burned in the time frame comparable to other reactive metal-fluoride composite powders. Most likely, initial fluorination of Si made it easier to initiate the reaction; however, it is expected that the laser energy was sufficient to rapidly heat Si particles, which did not sustain combustion once exposed to the room temperature air. It is thus suggested that the presence of reduced or partially reduced Bi accelerates both low- and high-temperature heterogeneous oxidation of Si, assisting in establishing and sustaining the particle flame.

As estimated by the CEA code (Figure S1), the high adiabatic flame temperatures volatilize a significant mass fraction of refractory silicon oxides formed during combustion of 50Si·50CoF_2_. In the case of 50Si·50BiF_3_, the mass fraction of volatile species might be higher because both bismuth and bismuth oxide vaporize easily. However, once bismuth is oxidized in the presence of a more reactive metal fuel, it is likely to be immediately reduced, serving as an oxygen shuttle. Thus, presence of Bi can effectively accelerate oxidation of Si, similarly to how it was suggested to accelerate oxidation of Al [20] and B [23]. When BiF_3_ serves as an oxidizer with different fuels, including Al, B and Si, gas phase products (including fluorides and oxyfluorides of the respective fuels) are favored a the high combustion temperatures. At the same time, the surface reaction may be governed by oxidation of Bi. Respectively, similar rates of combustion, with heterogeneous surface reactions yielding volatile products, are expected for all such composite materials. The rate of such reactions is expected to be controlled by diffusion of the oxidizer (oxygen) to the particle surface in all cases. This reasoning can explain comparable burn times observed for different composites, 50Al·50BiF_3,_ 50B·50BiF_3_ and 50Si·50BiF_3_ for similarly sized particles (Figure 15)

## 5. Conclusions

Preparing Si-based composites with BiF_3_ and CoF_2_ as oxidizers required a premilling step to refine the starting commercial Si powder in order to achieve nanoscale mixing between the material components. Preparation of well homogenized composites of Si with BiF_3_ could not be achieved without partial reaction of BiF_3_; however, no such reaction of CoF_2_ was observed. Upon heating, oxidation of Si occurs at lower temperatures than its fluorination when the added oxidizer is CoF_2_. Conversely, both oxidation and fluorination occurred nearly simultaneously and at a lower temperature with BiF_3_ as an oxidizer. The presence of reduced Co and Bi accelerated oxidation of Si upon its heating in an oxygen-containing environment.

Unlike elemental Si, prepared composite powders ignited readily when placed as a coating on an electrically heated filament. Ignition of the composite with BiF_3_ as an oxidizer occurred at consistently lower temperatures than that of the composite with CoF_2_. Results suggest that low-temperature fluorination and oxidation caused ignition for the composites with BiF_3_ as an oxidizer; apparent activation energy for reactions leading to ignition was close to 62 kJ/mol. For the composites with CoF_2_ as an oxidizer, it is likely that ignition is associated with Si fluorination.

Prepared composite powder with the BiF_3_ oxidizer ignited readily when passed through a CO_2_ laser beam in room temperature air. The measured particle burn times were nearly the same as those of similar composites combining BiF_3_ with such fuels as Al and B. Combustion products were mostly gaseous making it impossible to collect and analyze produced condensed species.

## Figures and Tables

**Table 1:** Estimated heats of reactions involving Si with oxides and fluorides of Co and Bi for stoichiometric and fuel-rich (50/50 wt.%) composites reacting in inert and oxidizing environments.

Composite Type			Heat of Reaction	
			kJ/g	kJ/cm^3^
Si with Bi_2_O_3_ and BiF_3_	Stoichiometric	3Si + 2Bi_2_O_3_ → 3SiO_2_ + 4Bi	−1.56	−11.25
		3Si + 4BiF_3_ → 3SiF_4_ + 4Bi	−1.05	−5.12
	50/50 wt.% (fuel rich) *	Si + Bi_2_O_3_ → Si + SiO_2_ + Bi	−0.85	−3.14
		Si + BiF_3_ → Si + SiF_4_ + Bi	−0.57	−1.84
		Added ambient oxidizer Si + Bi_2_O_3_ + O_2_ → SiO_2_ + Bi_2_O_3_	−15.6	−57.6
		Added ambient oxidizer Si + BiF_3_ + O_2_ → SiF_4_ + SiO_2_ + Bi_2_O_3_	−15.5	−50.22
Si with CoO and CoF_2_	Stoichiometric	Si + 2CoO → SiO_2_ + 2Co	−2.45	−12.32
		Si + 2CoF_2_ → SiF_4_ + 2Co	−1.28	−5.11
	50/50 wt.% (fuel-rich) *	Si + CoO → Si + SiO_2_ + Co	−1.45	−4.97
		Si + CoF_2_ → Si + SiF_4_ + Co	−0.60	−1.82
		Added ambient oxidizer Si + CoO + O_2_ → SiO_2_ + CoO	−14.63	−50.05
		Added ambient oxidizer Si + CoF_2_ + O_2_ → SiF_4_ + SiO_2_ + CoO	−14.46	−44.26

* For clarity of the concept, reactions are written without balancing reactants and products.

**Table 2:** Compositions of prepared silicon-metal fluoride reactive composites.

Milling Protocol	Material	Fluoride Volume (%)	Equivalence Ratio *ϕ*	Mass of Fluorine (%)	
				Prepared	Stoichiometric
Single stage	1-stage 50Si·50BiF_3_	30.4	12.6	10.7	19.9
Two stages	50Si·50BiF_3_				
	30Si·70 BiF_3_	50.5	5.4	15	
	50Si·50CoF_2_	34.3	6.9	19.6	34.2
	30Si·70CoF_2_	54.9	3.0	27.4	

**Table 3:** Compositions of the prepared Si·BiF_3_ composites obtained from the whole pattern refinement of the XRD traces.

Sample	Mass (%)				
	Si	Bi	BiF_3_	BiF_1.57_O_0.71_	BiF_1.98_O_0.51_
1-stage-50Si·50BiF_3_	55.2	1.7	22.4	9.7	11.0
50Si·50BiF_3_	45.6	12.0	16.6	17.3	8.5
30Si·70BiF_3_	26.6	16.4	24.9	20.8	11.3

## Supplementary Materials

The following are available online at https://www.mdpi.com/2079-4991/10/12/2367/s1, Figure S1: The calculated adiabatic flame temperature and the combustion products presented as mole fractions at a range of equivalence ratios for 50Si·50CoF2.
