# Supplementary material for: Preparation and Characterization of Silicon-Metal Fluoride Reactive Composites
Source: Nanomaterials (Basel). 2020 Nov 28;10(12):2367. doi: 10.3390/nano10122367 (PMC7760387; doi:10.3390/nano10122367)
Supplement: Supplementary file 1 [file nanomaterials-10-02367-s001.pdf]

nanomaterials

Supplementary Material

Preparation and Characterization of Silicon-Metal Fluoride Reactive Composites

Siva Kumar Valluri [1], Mirko Schoenitz [1] and Edward Dreizin [1,2,*]

1 O.H. York Department of Chemical and Materials Engineering, New Jersey Institute of Technology, Newark, NJ 07102, USA; sv476@njit.edu (S.K.V.); schoenit@njit.edu (M.S.)
2 High-Energy and Special Materials Research Laboratory, Tomsk State University, Tomsk 634050, Russia
* Correspondence: dreizin@njit.edu

Combustion in Air

The adiabatic flame temperature and predicted mole fraction of the products of $50Si \cdot 50CoF_2$ burning in air and calculated by NASA CEA code [1] are presented as a function of equivalence ratio in Figure S1. The calculations were performed for the constant pressure, 1 atm. The highest adiabatic flame temperature of 2750 K is predicted for the equivalence ratio range of 0.8–1.

In fuel-lean conditions ($\varphi << 1$) where lower adiabatic temperatures are expected, gaseous SiO was predicted to be the primary product with substantial gaseous F and Co species. The primary fluorinated product in the fuel-lean conditions is SiF_3. In fuel-rich conditions ($\varphi > 1$), representative of chemistry on the particle surface, considerable gasification of SiO_2 observed along with SiO. The primary fluorinated product is expected to be the thermodynamically stable SiF_4. Other fluorinated species such as HF and F are expected as well. Around the equivalence ratio of 0.8–1, the highest oxidation of silicon was observed with considerable gaseous and condensed phase SiO_2, along with products such as SiF_3 and SiF_4. The reduced cobalt could not be completely gasified across the range of equivalence ratios explored.

A similar calculation for $50Si \cdot 50BiF_3$ was not run because thermodynamic data for bismuth species are lacking. A significant portion of the combustion products for $50Si \cdot 50BiF_3$ is expected to be gaseous, facilitated by sufficiently high flame temperatures.

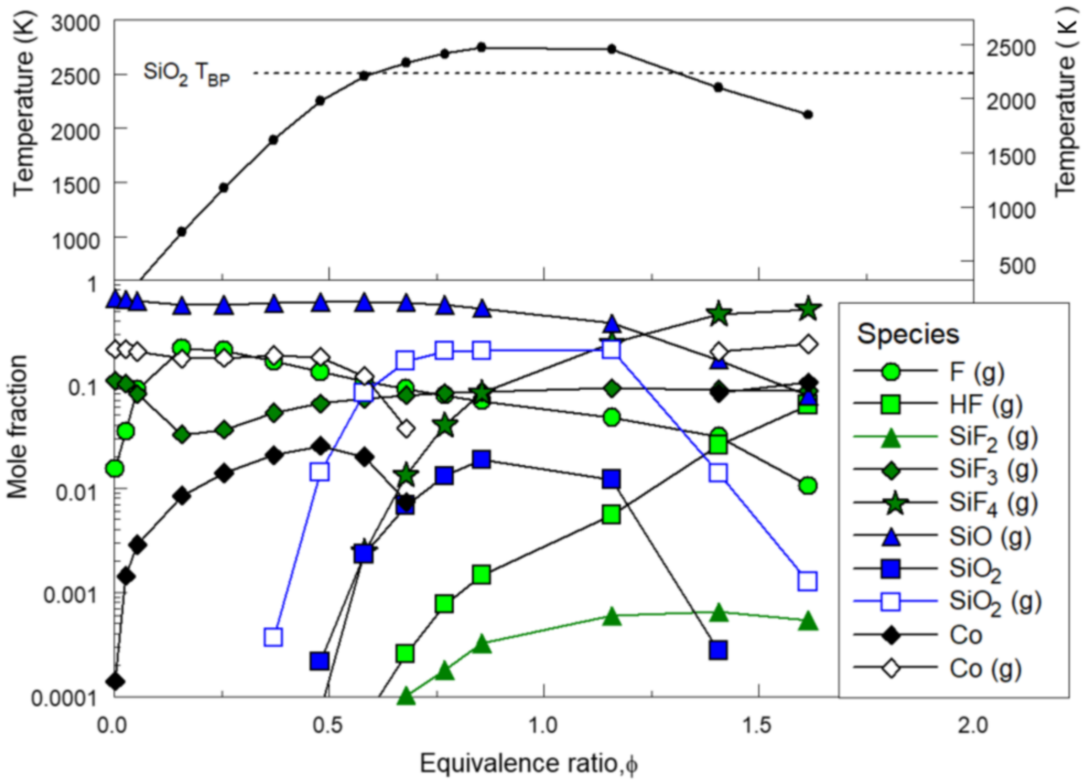

Figure S1. The calculated adiabatic flame temperature and the combustion products presented as mole fractions at a range of equivalence ratios for 50Si·50CoF_2.

References

1. McBride: B.J. and S. Gordon, *Computer Program for Calculation of Complex Chemical Equilibrium Compositions and Applications II. Users Manual and Program Description.* **1996**: NASA RP 1311, NASA Glenn Research Center, Cleveland, OH, USA.

Publisher's Note: MDPI stays neutral with regard to jurisdictional claims in published maps and institutional affiliations.

© 2020 by the authors. Licensee MDPI, Basel, Switzerland. This article is an open access article distributed under the terms and conditions of the Creative Commons Attribution (CC BY) license (http://creativecommons.org/licenses/by/4.0/).
